# Spongoid

**Published:** 1874-12

**Authors:** 


### SPONGOID.

This is a new preparation invented by J. F. French of New York; which is now being introduced to the dental profession. Is is an absorbent designed to be used instead of cotton, bibulous paper or spunk. From the experience we had with it we regard it as the best absorbent in use. It is in the form of a very thick blotting paper or felt, and may be cut into any required form for use. It is also prepared with a styptic property to be used in cases of hæmorrhage. As an absorbent it will be, we doubt not, eminently satisfactory to all who use it.

It can be obtained at the dental depots.

